# Probability Score for the Diagnosis of Periprosthetic Joint Infection: Development and Validation of a Practical Multi-analyte Machine Learning Model

**DOI:** 10.7759/cureus.84055

**Published:** 2025-05-13

**Authors:** Jim Parr, Van Thai-Paquette, Pearl Paranjape, Alex McLaren, Carl Deirmengian, Krista Toler

**Affiliations:** 1 Data Science and Machine Learning, Zimmer Biomet, Swindon, GBR; 2 Diagnostics Research and Development, Zimmer Biomet, Claymont, USA; 3 Orthopedic Surgery, University of Arizona College of Medicine - Phoenix, Phoenix, USA; 4 Orthopedic Surgery, Rothman Orthopaedic Institute, Philadelphia, USA; 5 Orthopedic Surgery, Thomas Jefferson University, Philadelphia, USA

**Keywords:** algorithmic analysis, alpha-defensins, artificial intelligence (ai), infection probability, machine learning, multianalyte assay, periprosthetic joint infection, pji diagnosis, synovial fluid analysis, total joint arthroplasty (tja)

## Abstract

Background and objective

The diagnosis of periprosthetic joint infection (PJI) relies on established criteria-based systems requiring interpretation and combination of multiple laboratory tests into scoring systems. In routine clinical care, clinicians implement these algorithms to diagnose PJI. Existing literature indicates suboptimal adoption and implementation of these criteria in clinical practice, even among experts. Recognizing the need for accurate PJI diagnosis through proper synthesis of multiple laboratory parameters, this study aimed to develop and validate a machine learning (ML) model that generates a preoperative PJI probability score based solely on synovial fluid (SF) biomarkers within 24 hours.

Materials and methods

A two-stage ML model was constructed using 104,090 SF samples from 2,923 institutions (2018-2024). First, unsupervised learning identified natural clusters in the data to label samples as “infected” or “not infected.” Then, these labels trained a supervised logistic regression model that generated PJI scores (0-100), categorizing cases as PJI positive (> 80), PJI negative (< 20), or equivocal (20-80).

The model incorporated 10 SF biomarkers: specimen integrity markers (absorbance at 280 nm, red blood cell count), inflammatory markers (white blood cell count, percentage of neutrophils, SF C-reactive protein), a PJI-specific biomarker (alpha-defensin), and microbial antigen markers (*Staphylococcus*, *Enterococcus*, *Candida*, and *Cutibacterium acnes*). Notably, culture results were excluded to allow for a 24-hour diagnosis. After splitting data into training (n = 83,272) and validation (n = 20,818) cohorts, performance was assessed against modified 2018 International Consensus Meeting criteria, including evaluation with probabilistically reclassified "inconclusive" cases.

Results

The ML model and resulting PJI score showed high diagnostic accuracy in the validation cohort. The PJI score achieved 99.3% sensitivity and 99.5% specificity versus the clinical reference before reclassification of inconclusive cases and 98.1% sensitivity and 97.6% specificity after probabilistic reclassification. With a disease prevalence of 20.7%, the positive predictive value reached 91.5% and the negative predictive value 99.5%. The model resolved 95% (1,363/1,442) of samples deemed inconclusive by the clinical standard. The analysis identified alpha defensin, percentage of neutrophils, and white blood cell count as the most influential model features. The model performed well in culture-negative infections.

Conclusions

The ML model and resulting PJI score demonstrated exceptional diagnostic accuracy by leveraging unsupervised SF biomarker pattern clustering. The model substantially reduced diagnostic uncertainty by definitively classifying most inconclusive cases, revealing their natural alignment with infected or non-infected patterns. This performance was achieved without SF culture results, enabling definitive diagnostic information within 24 hours based solely on biomarkers. The clinical significance demonstrates that an ML algorithm can match the diagnostic accuracy of complex clinical standards while transferring analytical complexity from clinicians to laboratories, minimizing the implementation gap that hinders current criteria-based approaches.

## Introduction

Periprosthetic joint infection (PJI) is a deeply concerning complication, affecting 1-2% of primary joint replacements and threatening patient outcomes [1]. Consequences of PJI include limb loss, death, and staggering economic costs for healthcare systems and patients [1,2]. Due to longstanding diagnostic challenges, academic bodies have advocated criteria-based systems to establish a standardized PJI definition [3,4]. Yet, as commonly seen in medicine, these definitions remain underutilized by clinicians. Many fail to order necessary tests, while others struggle to integrate diverse results and scoring systems accurately [5,6]. Even PJI experts unknowingly misapply specific thresholds or definitions [7-9]. Thus, there is a critical need for diagnostic tools that combine high accuracy with clinical usability, enabling all clinicians to leverage expert-level criteria effectively.

To address the need for a reliable, actionable preoperative diagnostic PJI tool, a machine learning (ML) model was previously developed to indicate the probability that PJI is present based on a standard set of synovial fluid (SF) biomarkers [10]. This previous work demonstrated the feasibility of an algorithm that utilized a pilot dataset. Building on this foundational work, the model was refined with a larger dataset and developed on a secured infrastructure using Azure Machine Learning (Microsoft Corp., Redmond, WA, USA) integrated with the laboratory information system (LIS). This integration enables an automated delivery of a PJI score alongside standard biomarker results within 24 hours of receipt of the patient's SF by the laboratory. The novel approach of a rapid ML-based PJI score delivered within a secured network presents advancements to the field beyond existing diagnostic methods.

This study aimed to evaluate the clinical performance of the ML-enabled PJI probability score and its ability to diagnose PJI against a clinical reference. Additionally, the study presents the performance of the PJI score in culture-negative PJI and inconclusive samples. Finally, the contributions of each biomarker within the model were elucidated by employing Shapley additive explanations (SHAP) [10-12].

## Materials and methods

Study design

We utilized real-world clinical SF biomarker results to create an ML-based PJI probability algorithm and assess its concurrence with a clinical reference definition of PJI (2018 ICM) [3]. A total of 137,691 samples from 2,923 institutions were tested at a single clinical laboratory between 2018 and 2024, and the data were deidentified in accordance with approval from the WIRB-Copernicus Group Institutional Review Board (approval number: 20150222). No institution accounted for more than 1.5% of the total sample set, and samples were tested from across the United States, ensuring a relevant and representative dataset. The study was conducted in accordance with the principles of the Transparent Reporting of Multivariable Prediction Model for Individual Prognosis or Diagnosis (TRIPOD+AI) reporting guidelines [13]. Productivity tools based on the GPT-4 architecture were utilized for copy-editing purposes only; those tools were not used to generate new content or concepts. This study has not been submitted to or evaluated by another journal.

Study data

SF samples from the hip or knee joint of adult patients (18 years or older) submitted for comprehensive PJI testing that underwent microbiological culture and assay for all 11 biomarkers were included. Samples with compromised integrity (red blood cells (RBC) > 1,000,000 cells/µl or absorbance at 280 nm wavelength (A280) outside of the range indicative of SF) were excluded [14]. We also excluded samples that were not cultured (Table 1). The total dataset of 104,090 samples was split into a training (80%, n = 83,272) and validation (20%, n = 20,818) cohort, with the most recent 20% allocated for validation. The training set was randomized and further split into a derivation (n = 66,617) and testing cohort (n = 16,655), where the algorithm was derived and tested prior to assessing performance with the previously unseen validation data.

**Table 1:** Inclusion and exclusion criteria A280: optical density (absorbance) measured at 280 nanometer wavelength, RBC: red blood cell, PJI: periprosthetic joint infection, SF: synovial fluid

Criteria		No. of samples
Inclusion criteria	Patients with a hip or knee arthroplasty	120,677
	Comprehensive SF testing performed for PJI	
	Adult patients (age ≥ 18)	
	Specimen integrity biomarkers RBC and A280	
Exclusion criteria	Specimen substantially diluted with blood (RBC > 1,000,000/μL) or specimen integrity compromised (A280 < 0.342 or A280 > 1.19)	10,445
	Not tested for culture	6,142
Total samples used in the study		104,090

Data collection

All SF specimens were submitted to a central clinical laboratory for comprehensive diagnostic testing (CD Laboratories, Zimmer Biomet, Towson, MD [15]). The available input biomarkers (i.e., ML features), all derived from the SF samples, included two specimen integrity biomarkers (A280 and RBC); three general inflammatory biomarkers (white blood cell count (WBC), percentage of polymorphonuclear cells (PMN%), and SF-C-reactive protein (SF-CRP)); one PJI-specific host-response biomarker (alpha defensin (AD)); and five direct microbial antigen detection biomarkers (two *Staphylococcus* targets (SPA and SPB), *Enterococcus* target (EF), *Candida* target (CP), and *Cutibacterium* acnes target (PAC)) (Table 2).

**Table 2:** Biomarker testing A280: optical density (absorbance) measured at 280 nanometer wavelength, WBC: white blood cell, PMN%: percentage of neutrophils, RBC: red blood cell, AD: alpha-defensin, SF-CRP: synovial fluid C-reactive protein, CR: C-reactive protein, EF: microbial antigen test *Enterococcus* species panel, CP: microbial antigen test *Candida* species panel, PAC: microbial antigen test *Cutibacterium acnes* panel, SPA: microbial antigen test *Staphylococcus* species panel A, SPB: microbial antigen test *Staphylococcus* species panel B, MID: microbial identification panel, ELISA: enzyme-linked immunosorbent assay

Test name	Biomarker	Test platform/technology used
Specimen integrity	A280	Spectrophotometry (A280)
WBC and RBC and differential	WBC	Cell counter: Sysmex® XN-2000 (automated) or AO Spencer 878784 (manual)
	PMN%	
	RBC	
AD	AD	ELISA
CRP	SF-CRP	Immuno-complex captured by antibody-coated latex particles detected on the Beckman Coulter AU480/AU680 clinical chemistry analyzer
MID	EF	Immunometric beads detected on Luminex 200®
	CP	
	PAC	
	SPA	
	SPB	

Machine learning model development

Two-Stage Model Training Pipeline

**Figure 1:**
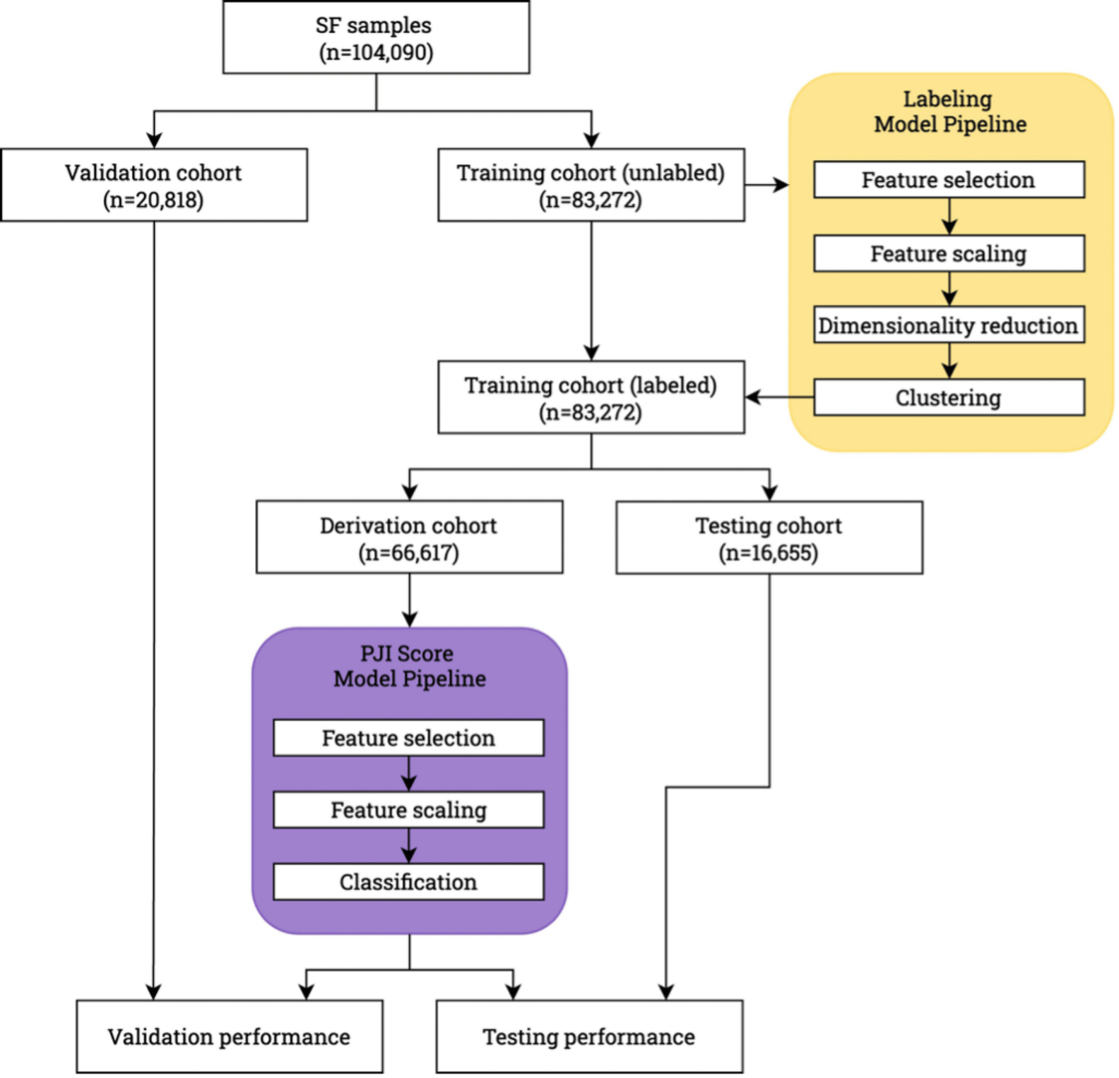
Two-stage model training pipeline that incorporated unsupervised and supervised methodologies. Feature selection refers to the set of input biomarkers used for each stage. Feature scaling refers to linear and non-linear transformations applied to the selected input biomarkers SF: synovial fluid, PJI: periprosthetic joint infection

There were three primary design requirements: (1) to create a ML model capable of predicting the probability of PJI independent of existing diagnostic labels using unsupervised ML techniques to identify intrinsic patterns in the data, (2) to enhance diagnostic performance on samples designated as inconclusive using a current diagnostic definition such as the 2018 International Consensus Meeting (ICM), and (3) to optimize clinical decision limits for intuitive decision making. These requirements led to the development of a two-stage model training pipeline incorporating unsupervised and supervised methodologies (Figure 1).

The two-stage model training pipeline was developed and implemented using Azure Machine Learning (Microsoft Corp., Redmond, WA, USA) and Python 3.10 (Python Software Foundation, Wilmington, DE, USA). The two primary components of this pipeline were the Labeling Model Pipeline and the PJI Score Model Pipeline (SynTuition™ Score, CD Laboratories, Towson, MD, USA), both implemented using scikit-learn (version 1.6.1, scikit-learn developers).

Labeling Model Pipeline

The Labeling Model Pipeline processed unlabeled training data and used unsupervised methods to infer infected/not infected labels.

As listed in the Data Collection section, 11 input biomarkers (i.e., features) were utilized. PMN% and A280 were scaled using a standard scaler. A Yeo-Johnson transformation [16] followed by a standard scalar was applied to RBC, WBC, SF-CRP, AD, EF, CP, PAC, SPA, and SPB. This combination of transformation and scaling normalized each feature so they shared a similar distribution and scale prior to unsupervised learning steps.

**Figure 2:**
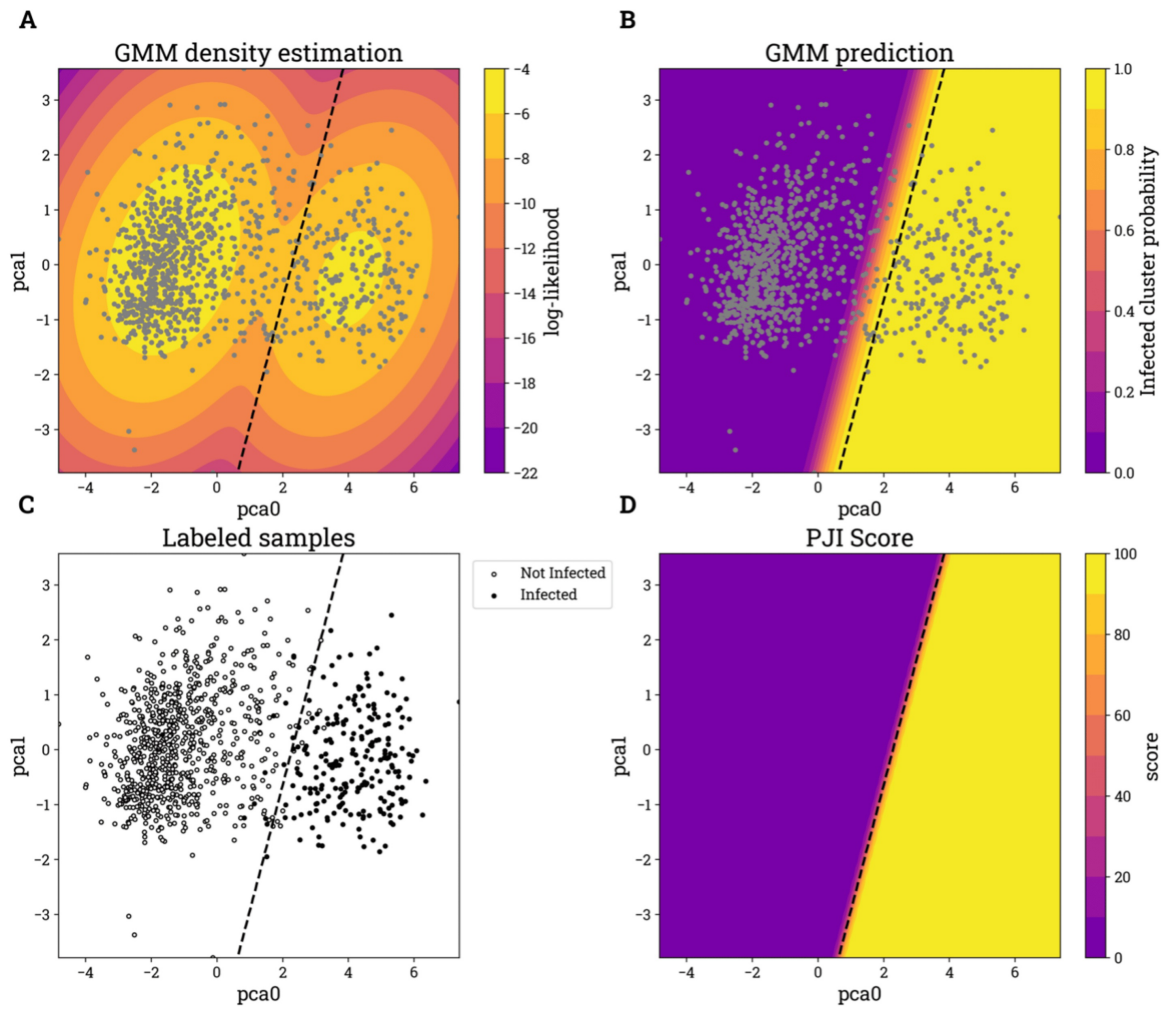
Visualization of unsupervised and supervised learning stages limited to two PCA components and a random subset of the training samples (n = 1,000). Each panel includes the 0.95 probability threshold, determined by the GMM prediction, as a dashed line. Training samples that exceeded a 0.95 probability threshold for the infected cluster were labeled infected, while all other samples were labeled not infected Panel A: The labeling model GMM density estimation. Panel B: The labeling model GMM prediction for the infected cluster probability. Note that the GMM tied covariance structure results in a linear separation between clusters. Panel C: The labeled subset of training samples. Panel D: The PJI score model logistic regression prediction. PCA: principal component analysis, GMM: Gaussian mixture model, PJI: periprosthetic joint infection

Principal component analysis (PCA) was applied to reduce dimensionality, resulting in five principal components preserving at least 85% of the total variance. The remaining components were discarded. A Gaussian mixture model (GMM) with a tied covariance structure and two mixture components was used to cluster the five principal components. The GMM, a probabilistic model, provided a density estimation (Figure 2, Panel A), which was used to assign samples to each cluster based on a probability threshold (Figure 2, Panel B). Two distinct clusters emerged, and high inflammatory biomarker values and a high culture positivity rate characterized samples in one of the two clusters. This cluster was assigned as the Infected cluster, and the other as not infected. Training samples that exceeded a 0.95 probability threshold for the Infected cluster were labeled Infected (Figure 2, Panel C), while all other samples were labeled not infected. Using a 0.95 probability threshold to assign labels as Infected yielded a proportion of Infected labels that appeared consistent with the anticipated disease prevalence [17,18].

PJI Score Model Pipeline

The PJI score Model Pipeline used supervised learning to classify labeled data and generate a probabilistic score for PJI.

Ten input features were used, including the same features from the Labeling Modeling Pipeline, except that SPA and SPB were replaced by their composite sum (SP Sum) to improve interpretability. Feature transformation and scaling were identical to those in the Labeling Model Pipeline. The SP Sum feature uses a Yeo-Johnson transformation followed by a standard scalar.

A logistic regression model was trained on 80% of the labeled training data (the derivation cohort), with performance evaluated on the remaining 20% (testing cohort). The model produced a probability score for the Infected class, which was subsequently scaled to an integer percentage, referred to as the PJI score (Figure 2, Panel D). A regularization term was incorporated into the logistic regression model training to mitigate the risk of complete separation. Complete separation can arise due to the perfect linear separation between labels imposed by the GMM, leading to instability in logistic regression training. A weak L2 regularization term (λ = 1.0) addressed this issue by ensuring robust parameter estimation [19].

Performance assessment

Clinical decision limits were set to classify samples into three categories. A PJI score above 80 indicates a high probability of infection (i.e., PJI positive), while a score below 20 indicates a low probability of infection (i.e., PJI negative). Scores between 20 and 80 are considered equivocal. These limits were chosen using receiver operator characteristic (ROC) curve analysis of the derivation set prior to validation (Table 3). These chosen decision limits ensure high sensitivity (99.1% at PJI score > 80) and specificity (99.4% at PJI score < 20) and align with physician feedback on preferred decision limits for clinical utility and decision-making. Classifying PJI scores into distinct clinical categories enabled the performance evaluation using an existing clinical reference.

**Table 3:** Impact of decision limits for a PJI positive diagnosis using the PJI score. Sensitivity and specificity are calculated based on the derivation cohort. Inconclusive diagnoses by clinical reference are not included PJI: periprosthetic joint infection

PJI score	Sensitivity (%)	Specificity (%)
90	98.9	99.7
80	99.1	99.7
70	99.2	99.6
60	99.2	99.6
50	99.3	99.6
40	99.3	99.5
30	99.3	99.5
20	99.4	99.4
10	99.5	99.3

Performance of the PJI score was compared to a modified 2018 ICM [3] criteria as the clinical reference, where SF-CRP was used in place of serum CRP in accordance with previous validation [20]. 2018 ICM was selected as the clinical reference due to its general acceptance as a valid definition of PJI, particularly in the United States, where the PJI score will be deployed. Since the PJI score is intended to be applied to specific, comprehensive SF test results to aid PJI diagnosis preoperatively, only preoperative SF components of the 2018 ICM were used to establish infection classification (Table 4).

**Table 4:** Criteria used to establish infection classification using the clinical reference. Infection was classified as follows: ≥ 6 points: PJI positive; ≤ 2 points: PJI negative; and 3-5 points: inconclusive WBC: white blood cell, S/CO: signal to cutoff, PMN%: percentage of neutrophils, SF-CRP: synovial fluid C-reactive protein, AD: alpha-defensin

Condition	Score
WBC > 3,000 cells/μL or AD S/CO ≥1.00	3 points
PMN% > 70%	2 points
SF-CRP > 4.45 mg/L	2 points
Positive culture result	2 points

Comparing the PJI score to the modified 2018 ICM definition provides a foundational reference point for clinicians. However, the presence of the inconclusive category prevents a true qualitative comparison. Simply discarding this category would lead to a flawed performance evaluation by ignoring the most difficult-to-predict cases. To better represent the performance of the PJI score, a probabilistic approach was used to establish rules for reclassifying inconclusive cases as “PJI positive” or “PJI negative” (Table 5). This method applies the combined specificity and sensitivity of each possible combination of biomarker results. It classifies the sample as positive if the combined specificity is higher than the combined sensitivity (i.e., the false positive rate is lower than the false negative rate) and as negative if the combined sensitivity is higher than the combined specificity (i.e., the false negative rate is lower than the false positive rate). Thus, all possible combinations of biomarkers leading to clinical reference scores of 3, 4, and 5 were evaluated and classified as PJI positive or PJI negative.

**Table 5:** Specificity and sensitivity of individual SF biomarkers used to develop reclassification rules. MID includes a positive microbial identification using a combination of the five microbial antigen detection biomarkers Culture [21,22], AD: alpha-defensin [15], MID: microbial identification panel [21], WBC: white blood cell [23], PMN%: percentage of neutrophils [23], SF-CRP: synovial fluid C-reactive protein [20]

Biomarker	Specificity	Sensitivity	False positive rate	False negative rate
Culture	99.5%	72.4%	0.0047	0.276
AD	97.4%	94.9%	0.026	0.051
MID	98.4%	73.8%	0.016	0.262
WBC	91.2%	80.6%	0.088	0.194
PMN%	84.3%	84.7%	0.157	0.153
SF-CRP	87.1%	86.1%	0.129	0.139

The PJI score was generated for every sample in the validation cohort, which had been previously set aside, and classified as PJI positive, PJI negative, or equivocal using the clinical decision limits. Sensitivity, specificity, positive predictive value (PPV), and negative predictive value (NPV) were assessed before and after reclassification of the inconclusive category. Results classed as equivocal by the PJI score for all performance assessments were treated as false.

Model interpretability

SHAP values were calculated for each training and validation sample to support the interpretability of the PJI score model. All SHAP values were computed using the Python shap package 0.46.0 (Scott Lundberg).

Statistical analysis

To ensure a sufficiently narrow confidence interval of ±2% and to meet the minimum a priori performance requirement of 95% sensitivity and specificity, the minimum required sample size was calculated to be at least 280 infected samples [24]. The number of infected samples in the validation set exceeded this minimum requirement. Due to their nonparametric distributions, continuous biomarker variables are presented as median (interquartile range, Q1 - Q3). The Mann-Whitney U test was utilized to assess differences between these variables. Cohen's d was then calculated based on the medians to determine the effect size, categorized as follows: very small (d < 0.2), small (0.2 ≤ d < 0.5), medium (0.5 ≤ d < 0.8), and large (d ≥ 0.8). The 95% confidence intervals (CI) for clinical performance metrics were calculated using the Wilson method. Differences between clinical subgroups were assessed using the two-proportion Z-test for comparisons between two subgroups or the Chi-square goodness-of-fit test for comparisons across multiple subgroups.

## Results

Baseline characteristics

A total of 104,090 SF samples were included in the study, with 83,272 included in the training cohort (66,617 derivation and 16,655 testing) and 20,818 in the validation cohort. Using the clinical reference, 18.3% of samples were PJI positive, 74.7% PJI negative, and 7.0% inconclusive. After reclassification of the inconclusive category, 41.0% of inconclusive samples were classified as PJI positive and 59.0% as PJI negative, resulting in 21.2% PJI positive and 78.8% PJI negative across all samples included in the study. Table 6 provides the split between training and validation cohorts.

**Table 6:** SF samples and characteristics between training (derivation and testing) and validation cohorts IQR: interquartile range, PJI: periprosthetic joint infection, SF: synovial fluid

Variable	Training (n = 83,272)	Validation (n = 20,818)
Time period	Jan 2018-Aug 2023	Aug 2023-Jul 2024
Organizations (count, samples per org)	2,548 (1-1,391)	1,699 (1-275)
Age (median (IQR))	66 (60-73)	68 (61-71)
Gender (samples, no. (%))		
Male	43,255 (51.9)	11,005 (52.9)
Female	39,976 (48.1)	9,792 (47.0)
Unknown	41 (0.0)	21 (0.1)
Joint type (samples, no. (%))		
Knee joint	76,562 (91.9)	19,168 (92.1)
Hip joint	6,710 (8.1)	1,650 (7.9)
Culture (samples, no. (%))		
Negative	72,108 (86.6)	17,959 (86.3)
Positive	11,164 (13.4)	2,859 (13.7)
Infection classification based on clinical reference (samples, no. (%))		
PJI negative	62,130 (74.6)	15,597 (74.9)
PJI positive	15,302 (18.4)	3,779 (18.2)
Inconclusive	5,840 (7.0)	1,442 (6.9)
Infection classification after reclassification of the inconclusive category (samples, no. (%))		
PJI negative	65,514 (78.7)	16,510 (79.3)
PJI positive	17,758 (21.3)	4,308 (20.7)

Machine learning model development

Labeling Model

The labeling model used 11 features to cluster and label the training samples. Of these, 18,635 (22.4%) were labeled Infected (Table 7). The cluster used to label samples as infected showed higher values across all biomarkers (i.e., features).

**Table 7:** Biomarker feature summary statistics for training samples labeled as not infected and infected using the labeling model IQR: interquartile range, PMN%: percentage of neutrophils, A280: optical density (absorbance) measured at 280 nanometer wavelength, CP: microbial antigen test *Candida* species panel, RBC: red blood cell, WBC: white blood cell, AD: alpha-defensin, EF: microbial antigen test *Enterococcus* species panel, SF-CRP: synovial fluid C-reactive protein, PAC: microbial antigen test *Cutibacterium acnes* panel, SPA: microbial antigen test *Staphylococcus* species panel A, SPB: microbial antigen test *Staphylococcus* species panel B

Feature	Not infected, median (IQR)	Infected, median (IQR)
	n = 64,637	n =18,635
PMN%	33.6 (24.1-46.5)	92.0 (85.2-95.3)
A280	0.59 (0.51-0.68)	0.80 (0.71-0.90)
CP	0.43 (0.38-0.5)	0.72 (0.63-0.84)
RBC	14,000 (5,000-45,000)	25,000 (10,000-68,000)
WBC	503 (277-972)	19,632 (7,948-40,035)
AD	0.08 (0.06-0.12)	2.49 (1.48-3.36)
EF	0.46 (0.42-0.50)	0.70 (0.62-0.83)
SF-CRP	0.9 (0.4-2.3)	16.0 (5.6-35.0)
PAC	0.10 (0.09-0.10)	0.10 (0.10-0.14)
SPA	0.56 (0.51-0.61)	0.86 (0.70-1.47)
SPB	0.50 (0.42-0.59)	1.16 (0.88-1.75)

PJI Score Model

The PJI score model used 10 biomarker features; nine of the 11 features plus SP Sum (derived from SPA and SPB) were trained on the labeling model's predicted labels. Summary statistics for the features and labels used for model development (training) and clinical performance evaluation (validation) were compared (Table 8). While most biomarker variables show statistical differences between the datasets, the overlapping interquartile ranges suggest these differences lack clinical significance. Cohen’s d values demonstrate that these differences have unimportant effect sizes.

**Table 8:** Feature summary statistics for the training (derivation and testing) and validation cohorts. Note that predicted labels are not required for the validation cohort IQR: interquartile range, PMN%: percentage of neutrophils, A280: optical density (absorbance) measured at 280 nanometer wavelength, CP: microbial antigen test *Candida* species panel, RBC: red blood cell, WBC: white blood cell, AD: alpha-defensin, EF: microbial antigen test *Enterococcus* species panel, SF-CRP: synovial fluid C-reactive protein, PAC: microbial antigen test *Cutibacterium acnes* panel, SPA: microbial antigen test *Staphylococcus* species panel A, SPB: microbial antigen test *Staphylococcus* species panel B, SP Sum: sum of microbial antigen *Staphylococcus* species panel A and B

Feature	Training, median (IQR)	Validation, median (IQR)	p-value	Cohen’s d	Effect size
PMN%	40.3 (26.9-67.3)	39.4 (25.5-66.9)	0.000	0.02	Very small
A280	0.63 (0.53-0.74)	0.62 (0.53-0.73)	0.435	0.01	Very small
CP	0.46 (0.39-0.59)	0.40 (0.35-0.48)	0.000	0.33	Small
RBC	16,000 (6,000-50,000)	16,000 (5,000-50,000)	0.134	0.00	Very small
WBC	717 (332-2,359)	645 (295-2,265)	0.000	0.04	Very small
AD	0.10 (0.07-0.42)	0.12 (0.09-0.39)	0.000	-0.06	Very small
EF	0.48 (0.43-0.58)	0.43 (0.37-0.57)	0.000	0.30	Small
SF-CRP	1.4 (0.5-5.1)	1.3 (0.4-4.8)	0.000	0.02	Very small
PAC	0.10 (0.09-0.10)	0.09 (0.08-0.11)	0.000	0.29	Small
SPA	0.58 (0.52-0.69)	0.56 (0.49-0.75)	0.000	0.07	Very small
SPB	0.54 (0.44-0.75)	0.48 (0.42-0.57)	0.000	0.25	Small
SP Sum	1.11 (0.97-1.45)	1.04 (0.92-1.31)	0.000	0.16	Very small
Predicted labels from labeling model, N (%)					
Not Infected	64,637 (77.6)	-			
Infected	18,635 (22.4)	-			

**Figure 3:**
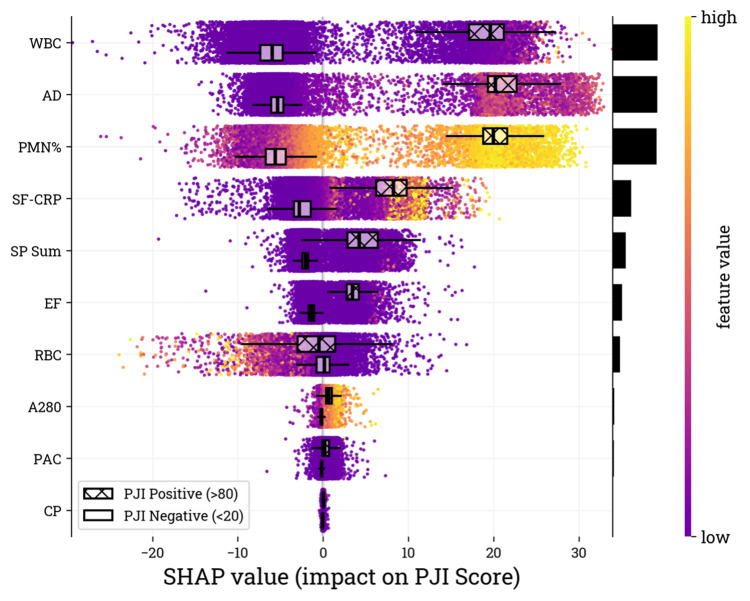
SHAP values for the validation cohort. The biomarker inputs (i.e., features) are listed on the y-axis in descending order of importance, with the features contributing the most at the top of the list as determined by the mean absolute SHAP value of the validation samples. Relative feature contribution is depicted by the black bars on the vertical axis. The SHAP value on the x-axis represents the feature contribution to the overall PJI score of each sample. Box plots are overlayed to show the distribution of SHAP values associated with the PJI positive and PJI negative subgroups based on the PJI score. Color, purple to yellow, represents the result value of the biomarker from lowest to highest, respectively SHAP: Shapley additive explanations, PJI: periprosthetic joint infection, A280: optical density (absorbance) measured at 280 nanometer wavelength, WBC: white blood cell, PMN%: percentage of neutrophils, RBC: red blood cell, AD: alpha-defensin, SF-CRP: synovial fluid C-reactive protein, EF: microbial antigen test *Enterococcus* species panel, CP: microbial antigen test *Candida* species panel, PAC: microbial antigen test *Cutibacterium acnes* panel, MID: microbial identification panel, ELISA: enzyme-linked immunosorbent assay

Analysis of SHAP values for each PJI score model input feature demonstrated that WBC, AD, and PMN% were the most influential parameters, with clear separation in SHAP values for PJI positive (> 80) and PJI negative (< 20) subgroups (Figure 3).

Clinical performance

**Figure 4:**
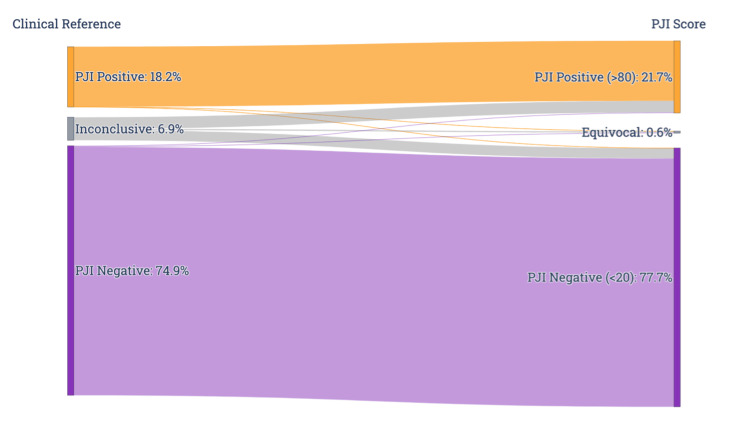
Sankey diagram showing the proportions of clinical reference and the PJI score categories for the validation cohort PJI: periprosthetic joint infection

The PJI score achieved a sensitivity of 99.3% (95% CI: 98.9% to 99.5%) and a specificity of 99.5% (95% CI: 99.4% to 99.6%) before the inconclusive category in the clinical reference was reclassified (Table 9). After reclassifying the inconclusive category, sensitivity was 98.1% (95% CI: 97.6% to 98.4%), and specificity was 97.6% (95% CI: 97.4% to 97.9%) (Table 10). Based on the observed disease prevalence of 20.7% in this population, PPV was 91.5% (95% CI: 90.7% to 92.3%), and the NPV was 99.5% (95% CI: 99.4% to 99.6%). The proportion of equivocal results was 0.6% (Figure 4). This performance is consistent across different subgroups (Table 11). Statistically significant differences in sensitivity and NPV were observed between the knee and hip subgroups. The hip subgroup was less than one-tenth the size of the knee subgroup.

**Table 9:** Confusion matrix for PJI score categories and the clinical reference for the validation cohort PJI: periprosthetic joint infection

PJI score	PJI positive (> 5)	PJI negative (< 3)	Inconclusive (3–5)	Total
PJI positive (> 80)	3,751	39	725	4,515
PJI negative (< 20)	14	15,521	638	16,173
Equivocal	14	37	79	130
Total	3,779	15,597	1,442	20,818

**Table 10:** Confusion matrix for PJI score categories and reclassified clinical reference for the validation cohort PJI: periprosthetic joint infection

PJI score	PJI positive	PJI negative	Total
PJI positive (> 80)	4,224	291	4,515
PJI negative (< 20)	54	16,119	16,173
Equivocal	30	100	130
Total	4,308	16,510	20,818

**Table 11:** Clinical performance on validation cohort subgroups based on the clinical reference with reclassification of the inconclusive category PPV: positive predictive value, NPV: negative predictive value

Group	Equivocal	Sensitivity	Specificity	PPV	NPV
Validation (n = 20,818)	0.6%	98.1%	97.6%	91.5%	99.5%
Gender					
Male (n = 11,005)	0.7%	98.0%	97.5%	91.1%	99.5%
Female (n = 9,792)	0.5%	98.2%	97.8%	92.0%	99.5%
p-value	0.098	0.636	0.204	0.299	0.579
Age					
< 50 (n = 856)	0.8 %	97.6%	97.1%	89.8%	99.4%
50-59 (n = 3,531)	0.7%	97.7%	98.1%	93.0%	99.4%
60-69 (n = 7,716)	0.6%	97.4%	97.6%	91.3%	99.3%
70-79 (n = 6,769)	0.6%	98.6%	97.6%	91.5%	99.6%
≥ 80 (n = 1,946)	0.6%	98.8%	97.2%	90.4%	99.7%
p-value	1.000	0.389	0.364	0.451	0.345
Joint type					
Knee (n = 19,168)	0.6%	98.3%	97.6%	91.6%	99.5%
Hip (n = 1,650)	0.9%	96.3%	97.5%	90.9%	99.0%
p-value	0.119	0.003	0.829	0.589	0.018

Clinical performance in the culture-negative subgroup

We evaluated the PJI score’s performance in diagnosing culture-negative samples. Of the 17,959 culture-negative samples in the validation cohort, 1,805 (10.1%) were predicted PJI positive, 16,040 (89.3%) PJI negative, and only 114 (0.6%) were predicted as equivocal. This classification strongly aligned with the clinical reference, with 98.9% sensitivity (95% CI: 98.1% to 99.4%) and 99.5% specificity (95% CI: 99.4% to 99.6%). After reclassifying the inconclusive category, sensitivity and specificity remained high at 97.2% (95% CI: 96.3% to 97.9%) and 97.6% (95% CI: 97.4% to 97.8%), respectively.

**Figure 5:**
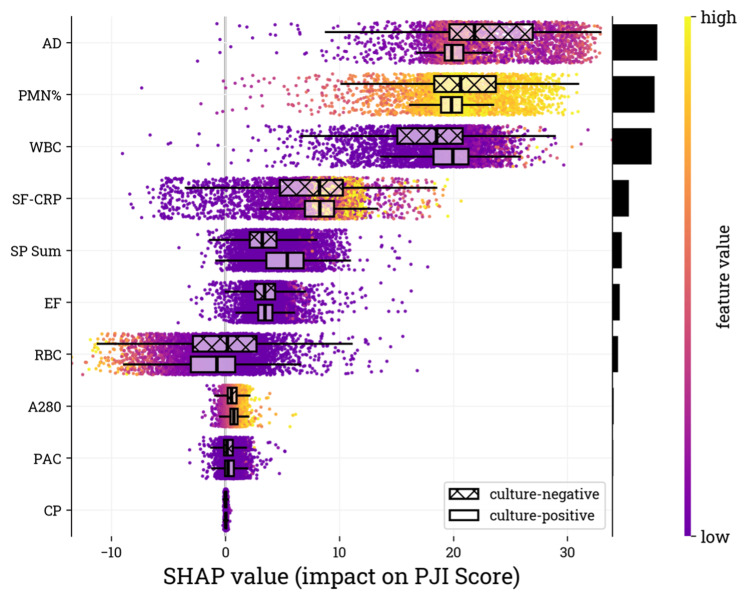
SHAP values for the PJI positive (> 80) subgroup in the validation cohort. The input biomarkers (i.e., features) are listed on the y-axis in descending order of importance, with the features contributing the most at the top of the list, as determined by the mean absolute SHAP value within the subgroup. Relative feature contribution is depicted by the black bars on the vertical axis. The SHAP value on the x-axis represents the feature contribution to the overall PJI score of each sample. Box plots are overlayed to show the distribution of SHAP values associated with culture-negative and culture-positive samples SHAP: Shapley additive explanations, PJI: periprosthetic joint infection, A280: optical density (absorbance) measured at 280 nanometer wavelength, WBC: white blood cell, PMN%: percentage of neutrophils, RBC: red blood cell, AD: alpha-defensin, SF-CRP: synovial fluid C-reactive protein, EF: microbial antigen test *Enterococcus* species panel, CP: microbial antigen test *Candida* species panel, PAC: microbial antigen test *Cutibacterium acnes* panel, MID: microbial identification panel, ELISA: enzyme-linked immunosorbent assay

Of particular interest are the samples indicative of culture-negative PJI. The SHAP values for the PJI positive (> 80) subgroup in the validation cohort illustrate the range of values for both culture-positive and culture-negative subsets (Figure 5). In this subgroup, AD emerged as the most influential feature. The culture-negative subset exhibits a broader range of SHAP values, consistent with broader biomarker distributions observed in this population. Despite this, the influence of each feature on the model's output remains similar between the culture-negative and culture-positive subsets.

Inconclusive subgroup analysis

**Figure 6:**
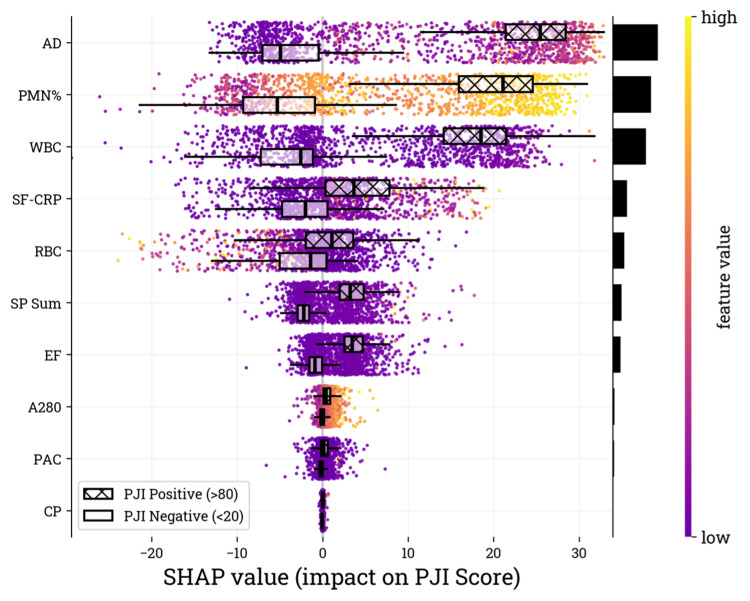
SHAP values for the clinical reference inconclusive subgroup in the validation cohort. The input biomarkers (i.e., features) are listed on the y-axis in descending order of importance, with the features contributing the most at the top of the list, as determined by the mean absolute SHAP value within the subgroup. Relative feature contribution is depicted by the black bars on the vertical axis. The SHAP value on the x-axis represents the feature contribution to the overall PJI score of each sample. Box plots are overlayed to show the distribution of SHAP values associated with the PJI positive and PJI negative subgroups based on the PJI score SHAP: Shapley additive explanations, PJI: periprosthetic joint infection, A280: optical density (absorbance) measured at 280 nanometer wavelength, WBC: white blood cell, PMN%: percentage of neutrophils, RBC: red blood cell, AD: alpha-defensin, SF-CRP: synovial fluid C-reactive protein, EF: microbial antigen test *Enterococcus* species panel, CP: microbial antigen test *Candida* species panel, PAC: microbial antigen test *Cutibacterium acnes* panel, MID: microbial identification panel, ELISA: enzyme-linked immunosorbent assay

Using the clinical reference, we evaluated the PJI score’s ability to predict preoperatively classified samples as inconclusive. The PJI score classified 94.5% of these samples as either PJI positive or PJI negative, with AD being the most influential feature (Figure 6). Only 79 out of 1,442 samples in this subgroup were categorized as equivocal (PJI score between 20 and 80).

Notably, 1,356 of the 1,442 inconclusive samples were also culture-negative, meaning they would have remained ambiguous even after culture results became available. With the PJI score, only 70 (5.2%) of these ambiguous samples remained equivocal, while the remaining 1,286 could be classified as either PJI positive or PJI negative.

## Discussion

In this study, we only developed a PJI score based on preoperative SF biomarkers. We found it to be highly predictive of the presence of PJI, with sensitivity and specificity exceeding 99% prior to reclassification of the inconclusive cohort. Even after reclassifying and including these challenging samples, sensitivity and specificity exceeded 97%, and the PJI score reduced the number of ambiguous diagnoses from 23.5% to 0.6% compared to physician interpretation of PJI biomarker results [5]. This performance remained consistent across different subgroups, though sensitivity was slightly lower for hips compared to knees (96.3% vs. 98.3%), likely due to the smaller sample size in the hip cohort.

The PJI score was developed using a complete set of contemporaneously performed biomarkers from previously validated clinical diagnostic tests. We implemented a two-stage training pipeline to create a classification model independent of existing diagnostic definitions. In the first stage, the labeling model pipeline applied unsupervised learning techniques, including PCA and GMM, to reduce dimensionality and cluster the data. This process identified two natural clusters, which were then used to assign labels, infected or not infected, to each specimen. In the second stage, the PJI score model pipeline used logistic regression to generate a probability score based on these labels. Clinical decision limits were established by ROC curve analysis and by incorporating user preference for decision limits near the extremes of 0 and 100. These limits were then used to categorize the PJI score as PJI positive (> 80), PJI negative (< 20), or equivocal (20-80).

Assigning infected labels using the GMM prediction threshold of 0.95 resulted in a disease prevalence of 22.4% within the training cohort, which closely aligns with the published prevalence of PJI in hip and knee arthroplasty, ranging from 17% to 25% [17,18]. Lowering the probability threshold (e.g., 0.5) would have classified more samples as infected, leading to a more sensitive but less specific algorithm. A lower threshold may be preferable when evaluating performance against a more sensitivity-focused clinical reference, such as the European Bone and Joint Infection Society (EBJIS) criteria, indicating a higher disease prevalence in the population [25].

The performance of our model was compared to a modified 2018 ICM criteria, which has gained popularity but is not the only clinical reference definition available. Efforts were made to reclassify inconclusive cases to minimize bias when evaluating performance, yet the reported performance remains sensitive to the reclassification methodology employed. As an alternative reclassification methodology, reclassifying all samples with a modified ICM score ≥ 4 as PJI positive biases the PJI positive category, resulting in a sensitivity of 91.6% and specificity of 98.9%, and alternatively, reclassifying all samples with an ICM score ≤ 4 as PJI negative biases the PJI negative category, resulting in a sensitivity of 96.0% and specificity of 98.3%. While we believe the probabilistic approach is more rigorous and objective, the results of this alternative approach confirm that the PJI score performs robustly regardless of the inconclusive classification method applied. We further maintain that the imperfection associated with any reclassification approach may very well result in an underestimated performance of the PJI score after the clinical reference. Inconclusive samples are reclassified.

Three studies have reported the development and performance of ML models for diagnosing PJI. Kuo et al. [26] published a small study (N = 323) on an ML model incorporating serum biomarkers, culture results, SF biomarkers, histology, and purulence. This model's performance was compared to the 2018 ICM criteria, and patient-specific explanations for diagnosing PJI were provided. However, the model was trained using infected and non-infected samples based on the ICM criteria, which is suboptimal since its accuracy is validated using the same criteria used to train it. The current study’s PJI score leveraged a huge dataset. It did not utilize clinical data such as purulence or histology, allowing for implementation by the laboratory conducting the clinical testing.

Furthermore, instead of training and validating the model with the same criteria, this study assigned labels to samples based on unsupervised natural clustering, followed by clinical reference standard validation. The PJI score uses simple logistic regression and SHAP explanations, making the results easily interpretable for clinicians. The other two studies, by Tao et al. [27] and Li et al. [28], applied deep learning methods for PJI diagnosis. Tao et al. used convolutional networks to identify pathological sections of PJI patients, while Li et al. utilized CT images for PJI diagnosis. These deep learning models face the "black box" problem [29], where the decision-making process is not transparent, unlike the PJI score, which offers clear and interpretable results for clinicians.

Opportunities for improved decision making

While various clinical definitions for PJI diagnosis exist, implementing them in practice remains challenging. The use of the 2018 ICM criteria, for instance, is susceptible to human error [5,30]. Using this ML-enabled PJI score automates the process of ordering, compiling, and scoring test results, thus reducing the risk of human error. Additionally, some scoring criteria, such as the 2018 ICM, rely on culture results before a diagnosis can be conclusively made, leading to delays in treatment decisions. In contrast, the PJI score can be calculated within 24 hours of receipt by the laboratory, without the need for culture results.

The presence of culture-negative results complicates diagnosis and is a particular concern in chronic and late-presenting cases [31]. The performance of the PJI score within the culture-negative subgroup was > 97% for both sensitivity and specificity (supplementary appendix, culture-negative subgroup analysis). The PJI score also showed similar biomarker contributions for culture-negative and culture-positive subsets, making it a strong candidate for aiding decision-making when identifying culture-negative PJI.

This study found that most samples classified as inconclusive using a clinical definition/score were also culture-negative. These cases experience delays due to waiting for culture results, and the diagnosis often remains unclear even after results are available. For such cases, a diagnosis must frequently be made using non-microbiological criteria, further delaying diagnosis [25]. This delay and ambiguity may increase the likelihood of misdiagnosis and increase the clinical and economic burden of PJI [2,32,33]. The authors of the EBJIS criteria for PJI state that failure to diagnose PJI correctly can lead to insufficient treatment and serious consequences. On the other hand, overdiagnosis can result in unnecessary invasive procedures [4]. For patients with inconclusive classification by the clinical reference and culture-negative results, the PJI score significantly reduces ambiguity, providing 95% of these patients with a PJI positive (> 80) or PJI negative (< 20) classification within 24 hours.

Clinical definitions of PJI rapidly evolve, but adoption into clinical practice can be slow [34]. This PJI score is ML software integrated with a standard LIS, which can be periodically re-evaluated and rapidly updated to stay aligned with the latest definitions. This adaptability can help accelerate clinicians' adoption of new definitions, ensuring faster implementation and improved patient care.

Limitations

This study has several limitations that warrant consideration. First, as a real-world data study, we did not have access to clinical data or treatment decisions, which limits our ability to directly assess how the diagnostic findings influenced patient management and outcomes. This analysis did not consider patient-specific factors such as readmissions, comorbidities, immune deficiencies, and prior antibiotic treatments. While these variables may be helpful for stratification of results, the large dataset used in this study reduces the potential impact of these factors on overall performance.

Second, while the dataset used was derived from a single laboratory, a notable strength is the well-controlled nature of the data, ensuring specimen and data integrity on a contemporaneously tested set of biomarkers. However, external data validation would be necessary to confirm broader applicability to other laboratories.

Third, the model used features not available from other laboratories, which include five direct microbial antigen detection biomarkers. Without these biomarkers, the model would be significantly weaker, so if modified to include only the widely available biomarkers, the PJI score would still be highly advantageous compared to current PJI definitions.

Fourth, the study focused exclusively on hip and knee arthroplasties, excluding data from other joints such as the shoulder, which may harbor different infectious organisms and exhibit distinct inflammatory responses. Expanding the dataset to include additional joints in future research could provide a more comprehensive model performance evaluation. Notably, the 2018 ICM definition of PJI is only established for use with hip and knee arthroplasties, so incorporating other joints into the same study would require definitions validated for those joints.

Fifth, some PJIs can be diagnosed with minimal investigations, and not all diagnostic tests are always necessary. However, a more extensive diagnostic approach can lead to a more accurate diagnosis in some cases [32]. This study emphasizes the importance of preoperative diagnostic rigor. Future work could explore how the model performs in cases with fewer diagnostic tests.

Finally, the model was trained based on the disease prevalence observed in the current clinical setting. Variations in PJI prevalence across different clinical settings may impact the model’s predictive values. However, the strength of an ML model lies in its ability to adapt and fine-tune to diverse and changing environments quickly. Future research should explore this adaptability by incorporating more varied clinical cohorts.

## Conclusions

The ML model and resulting PJI score demonstrated exceptional diagnostic accuracy by leveraging unsupervised SF biomarker pattern clustering. The model substantially reduced diagnostic uncertainty by definitively classifying most inconclusive cases, revealing their natural alignment with infected or non-infected patterns. This performance was achieved without using SF culture results, enabling definitive diagnostic information within 24 hours based solely on biomarkers. The clinical significance demonstrates that an ML algorithm can match the diagnostic accuracy of complex clinical standards while transferring analytical complexity from clinicians to laboratories, minimizing the implementation gap that hinders current criteria-based approaches.
